# Acetylcholinesterase and monoamine oxidase-B inhibitory activities by ellagic acid derivatives isolated from *Castanopsis cuspidata* var. *sieboldii*

**DOI:** 10.1038/s41598-021-93458-4

**Published:** 2021-07-06

**Authors:** Jong Min Oh, Hyun-Jae Jang, Myung-Gyun Kang, Soobin Song, Doo-Young Kim, Jung‑Hee Kim, Ji-In Noh, Jong Eun Park, Daeui Park, Sung-Tae Yee, Hoon Kim

**Affiliations:** 1 Department of Pharmacy, and Research Institute of Life Pharmaceutical Sciences, Sunchon National University, Suncheon, 57922 Republic of Korea; 2 Natural Medicine Research Center, Korea Research Institute of Bioscience and Biotechnology, Cheong-ju si, Chungcheongbuk-do 28116 Republic of Korea; 3 Department of Predictive Toxicology, Korea Institute of Toxicology, Daejeon, 34114 Republic of Korea

**Keywords:** Biochemistry, Computational biology and bioinformatics, Drug discovery

## Abstract

Among 276 herbal extracts, a methanol extract of *Castanopsis cuspidata* var. *sieboldii* stems was selected as an experimental source for novel acetylcholinesterase (AChE) inhibitors. Five compounds were isolated from the extract by activity-guided screening, and their inhibitory activities against butyrylcholinesterase (BChE), monoamine oxidases (MAOs), and β-site amyloid precursor protein cleaving enzyme 1 (BACE-1) were also evaluated. Of these compounds, 4′-*O*-(α-l-rhamnopyranosyl)-3,3′,4-tri-*O*-methylellagic acid (**3**) and 3,3′,4-tri-*O*-methylellagic acid (**4**) effectively inhibited AChE with IC_50_ values of 10.1 and 10.7 µM, respectively. Ellagic acid (**5**) inhibited AChE (IC_50_ = 41.7 µM) less than **3** and **4**. In addition, **3** effectively inhibited MAO-B (IC_50_ = 7.27 µM) followed by **5** (IC_50_ = 9.21 µM). All five compounds weakly inhibited BChE and BACE-1. Compounds **3**, **4**, and **5** reversibly and competitively inhibited AChE, and were slightly or non-toxic to MDCK cells. The binding energies of **3** and **4** (− 8.5 and − 9.2 kcal/mol, respectively) for AChE were greater than that of **5** (− 8.3 kcal/mol), and **3** and **4** formed a hydrogen bond with Tyr124 in AChE. These results suggest **3** is a dual-targeting inhibitor of AChE and MAO-B, and that these compounds should be viewed as potential therapeutics for the treatment of Alzheimer’s disease.

## Introduction

Cognitive dysfunctions, such as learning, memory, processing information speed, visual perception, mental flexibility, and persistent attention-deficit dysfunctions, are the primary symptoms of Alzheimer's disease (AD). Acetylcholine (ACh) regulates cognitive functions, especially learning and memory, via neurotransmission and is synthesized from choline and acetyl co-enzyme A in presynaptic neurons and then released into the synaptic gaps. Acetylcholinesterase (AChE) terminates the ACh-mediated neurotransmission and is mostly found in neurons^1,2^. The therapeutic efficacies of AChE inhibitors in AD have been shown to be due to augment synaptic ACh levels in the cerebral cortex and improve cholinergic transmissions^3^. In AD, levels of ACh are low and cause central nervous system (CNS) disorders, which are characterized by gradual declines in cognition, memory, and cognitive functions. Currently, AChE inhibitors approved by the FDA for the treatment of AD include donepezil, galantamine, and rivastigmine. Like AChE, butyrylcholinesterase (BChE) also importantly contributes to cholinergic neurotransmission. BChE is present in glial cells, hippocampus, and the temporal nerve cortex, and is involved in cognitive function. BChE has less substrate specificity than AChE, but both enzymes effectively hydrolyze ACh^2^.

Importantly, the formation and aggregation of β-amyloid peptide (Aβ) are associated with the hydrolysis of amyloid precursor protein (APP) by β-site amyloid precursor protein cleaving enzyme 1 (BACE-1; β-secretase-1), and since the anionic site of AChE is involved in Aβ aggregation, studies on dual AChE and BACE-1 inhibitors are being actively pursued^4^.

On the other hand, monoamine oxidases (MAOs) are involved in the pathways leading to catecholamine and 5-hydroxytryptamine inactivation, and thus, MAOs are recognized targets in diseases associated with these pathways. Furthermore, selective MAO-A inhibitors have anti-depressant activity, and selective MAO-B inhibitors are recognized developmental targets for the treatment of AD and Parkinson's disease (PD)^5^. Irreversible MAO-B inhibitors such as rasagiline and selegiline are used for the treatment of PD^6–8^, alongside levodopa, dopamine agonists, and catechol-*O*-methyltransferase inhibitors.

Recently, a multi-targeting treatment strategy was devised to target MAO-B and AChE, and it has been reported that MAO and AChE inhibitors can improve the cognitive functions and relieve symptoms in AD by increasing monoamine and choline ester levels^9^.

During our on-going efforts to identify potent natural inhibitors of MAO-A, MAO-B, and AChE in a herbal extract library, we found that rhamnocitrin isolated from the leaves of *Prunus padus* var. *seoulensis*, potently and selectively inhibits human MAO-A^10^, and that calycosin isolated from *Maackia amurensis* potently and selectively inhibits human MAO-B^11^. In this study, we screened *Castanopsis cuspidata* var. *sieboldii* as a potent AChE inhibiting herbal source, and isolated AChE inhibitors by activity-guided fractionation.

*C. cuspidata* is an evergreen broad-leaved tree that is widely distributed in western Japan^12^, and has been reported to contain galloyl shikimic acid^13^, ellagitannins^14^, and dehydrodiallic acid, cretanin, chesnatin, chestanin^15^, galloyl ester triterpenoid, and hexahydorxydiphenic acid conjugated triterpenoid^16^. Furthermore, extract of *C. cuspidata* has antioxidant^17,18^, anticancer, and anti-inflammatory^18^, and anti-fungal^19^ properties.

In this study, *C. cuspidata* was selected as an AChE inhibitor resource from a library of herbal extracts, five compounds were isolated and identified in an extract of the stems of the plant. Their inhibitory activities against AChE, BChE, MAO-A, MAO-B, and BACE-1 were evaluated, and kinetic and reversibility studies, cytotoxicity tests, in silico pharmacokinetics, and docking simulations were performed to identify novel candidate compounds for the treatment of AD and PD.

## Materials and methods

### General experimental procedures

^1^H and ^13^C NMR spectroscopic data were recorded using JEOL ECZ500R and JEOL ECA600 (JEOL, Tokyo, Japan) instruments, respectively. HR-ESI-MS data were acquired using an ACQUITY UPLC I-Class/Vion IM-QTOF system (Waters, Milford, MA, USA) coupled with ACQUITY BEH C_18_ column (Waters, 2.1 × 100 mm, 1.7 µm). Preparative HPLC was performed using a YMC K-Prep LAB 300 (YMC, Kyoto, Japan) equipped with a DAD-50-700S column (50.0 × 700 mm) packed with YMC ODS AQ HG resin (YMC, 10 µm, 500 g) and a Gilson HPLC system (GX271, a 321 pump, and a 172 diode array detector, Gilson, Middleton, WI, USA) coupled with an Acclaim Polar Advantage II C_18_ column, 250 × 21.2 mm, 5 µm (Thermo Fisher Scientific, Waltham, MA, USA), respectively.

### Plant material and the isolation of compounds 1–5

A methanol extract of the stems of *C. cuspidata* var. *sieboldii* was provided by the Korean Plant Extract Bank at the Korea Research Institute of Bioscience and Biotechnology (KRIBB, Daejeon, Korea) under agreement on Jan 4, 2020. The plant was identified by Dr. Gwanpil Song, and collected from Aewol-eup, Jeju-do, Korea in 2016. A voucher specimen (KRIB 0084147) was deposited at the KRIBB herbarium. All the experimental protocols adhered to the relevant ethical guidelines/regulations on the usage of plants. The extract (50 g) was separated by preparative reverse phase column chromatography eluting using a H_2_O/MeOH gradient (0–15 min, 5% MeOH; 15–110 min, 5 → 20% MeOH; 110–140 min, 20 → 50% MeOH; 140–150 min, 50 → 100% MeOH; and 150–180 min, 100% MeOH) to obtain ten fractions (1–10) using the YMC K-Prep LAB300 unit (YMC, DAD-50-700S, flow rate: 100 mL/min). Of these fractions, two (CCS8 and CCS9) identified by bioactivity screening for AChE inhibition were purified by preparative HPLC. CCS8 (2.9 g) was subjected to reverse phase column chromatography using a YMC K-Prep LAB300 and a solvent gradient (0 → 100% MeOH over150 min at 100 mL/min) to yield ten sub-fractions (CCS8-1–CCS8-10). Sub-fraction CCS8-1 (179.6 mg) was purified by preparative HPLC (Acclaim Polar Advantage II, 15 → 20% ACN over 50 min at 15 mL/min) to obtain compound **1** (62.1 mg). Compound **2** (4.5 mg) was obtained from sub-fraction CCS8-5 (297.1 mg) by preparative HPLC (Acclaim Polar Advantage II, 20 → 40% ACN over 50 min at 15 mL/min) and compound **3** (9.3 mg) was isolated from sub-fraction CCS8-6 (30.9 mg) by preparative HPLC (Acclaim Polar Advantage II, 25 → 100% ACN over 65 min at 15 mL/min). CCS-9 (3.6 g) was separated by preparative HPLC, YMC K-Prep LAB300 (YMC, DAD-50-700S, 40% ACN 0–10 min; 40 → 80% ACN 10–90 min; 80 → 100% ACN 90–95 min; 100% ACN 95–120 min at 100 mL/min) to produce ten sub-fractions (CCS9-1–CCS9-10). Compound **5** (6.8 mg) was obtained from sub-fraction CC-9–1-2 (64.7 mg) by preparative HPLC (Acclaim Polar Advantage II, 40 → 60% ACN over 75 min at 15 mL/min) and compound **4** (4.2 mg) was purified from sub-fraction CC-9–1-4 (50.7 mg) by preparative HPLC (Acclaim Polar Advantage II, 55 → 65% ACN over 45 min at 15 mL/min).

### Chemicals and enzymes

Recombinant human MAO-A and MAO-B, kynuramine, benzylamine, toloxatone, lazabemide, AChE (Type VI-S from *Electrophorus electricus*), BChE (equine serum), acetylthiocholine iodide (ATCI), butyrylthiocholine iodide (BTCI), 5,5′-dithiobis(2-nitrobenzoic acid) (DTNB), tacrine, donepezil, and the BACE-1 activity detection kit were purchased from Sigma-Aldrich (St. Louis, MO, USA). Clorgyline and pargyline were obtained from BioAssay Systems (Hayward, CA, USA).

Roswell Park Memorial Institute-1640 medium (RPMI-1640), Dulbecco’s Modified Eagle Medium (DMEM), fetal bovine serum (FBS), and penicillin/streptomycin solution were purchased from Hyclone Laboratories (San Ramon, CA, USA). The cell counting kit-8 (CCK-8) and dimethyl sulfoxide (DMSO) were obtained from Dojindo Laboratories (Kumamoto, Japan) and Sigma-Aldrich, respectively.

### Enzyme assays

AChE and BChE inhibitory activities were measured after preincubating enzymes with inhibitors for 15 min before adding substrates (ATCI and BTCI, respectively) and DTNB. AChE activities were assayed as previously described^20^, with slight modification^21,22^. Reactions were performed using ~ 0.2 U/mL of enzyme in the presence of 0.5 mM substrate and 0.5 mM of DTNB in 0.5 mL reaction mixtures, which were continuously monitored for 15 min at 412 nm. MAO-A and MAO-B activities were determined using a continuous spectrophotometric method, as described previously^23,24^. The K_m_ values of MAO-A for kynuramine and of MAO-B for benzylamine were 0.040 and 0.17 mM, respectively. The concentrations of kynuramine (0.06 mM) and benzylamine (0.3 mM) used were 1.5 times and 1.8 times K_m_ values, respectively. Reaction rates are expressed as changes in absorbance per min. BACE-1 assays were performed using a β-secretase (BACE-1) activity detection kit at excitation and emission wavelengths of 320 and 405 nm, respectively, using a fluorescence spectrometer (FS-2, Scinco, Seoul, Korea), after reaction for 2 h at 37 °C with 7-methoxycoumarin-4-acetyl-[Asn670,Leu671]-amyloid β/A4 protein fragment 667–676-(2,4-dinitrophenyl)Lys-Arg-Arg amide trifluoroacetate as substrate^25^.

### Analysis of inhibitor reversibility

The reversibilities of AChE and MAO-B inhibitions by **3**, **4**, and **5** were investigated by dialysis at concentrations of ~ 2 × IC_50_ values; tacrine and donepezil were used as reference AChE and BChE inhibitors^22,26^, and lazabemide and pargyline were used as reference reversible and irreversible MAO-B inhibitors, respectively^21^. After preincubating three compounds or reference inhibitors with enzymes for 15 min, residual activities of undialyzed (A_U_) and dialyzed (A_D_) samples were expressed as percentages of those of non-inhibitor treated controls. Reversibilities were assessed using A_U_ and A_D_ values and compared with those of reference compounds.

### Inhibitory activities and enzyme kinetics

Inhibitions of AChE, BChE, MAO-A, MAO-B, and BACE-1 by compounds **1** to **5** were initially investigated at a concentration of 10 µM^27,28^. IC_50_ values of the five compounds and the reference compounds (tacrine and donepezil for AChE and BChE, toloxatone and clorgyline for MAO-A, lazabemide and pargyline for MAO-B, and quercetin for BACE-1) were determined. Kinetic parameters, inhibition types, and K_i_ values of the most potent AChE inhibitors (**3** and **4**) and MAO-B inhibitors (**3** and **5**) were analyzed. Kinetics of AChE inhibitions by **3**, **4**, and **5** and of MAO-B inhibitions by **3** and **5** were investigated at five different substrate concentrations (0.05, 0.10, 0.20, 0.50, and 1.0 mM for AChE and 0.0375, 0.075, 0.15, 0.3, and 0.6 mM for MAO-B) in the absence or presence of each inhibitor at concentrations of ~ 0.5 × , 1.0 × , and 2.0 × their IC_50_ values. Inhibitory patterns and K_i_ values were determined using Lineweaver–Burk (LB) plots and secondary plots of LB slopes.

### Cytotoxicity test

Madin-Darby canine kidney (MDCK) cells and human acute promyelocytic leukemia (HL-60) cells were obtained from the Korean Cell Line Bank (Seoul, Korea). MDCK cells were cultured in DMEM, and HL-60 cells in RPMI-1640 medium containing 10% FBS, 1% penicillin/streptomycin, and 0.1% 2-mercaptoethanol. Cultures were maintained at 37 °C under 5% CO_2_, and media were changed every two days.

Cell viabilities were determined using the CCK-8 assay^29^. Briefly, MDCK cells or HL-60 cells were resuspended at 1 × 10^5^ or 3 × 10^5^ cells/mL and suspensions (100 µL) were added to wells of a 96-well plate and incubated in 5% CO_2_ atmosphere at 37 °C for 24 h. After incubation, 100 µL of each medium was treated with compounds at 1, 3, 10, 30, or 50 µM and incubated in 5% CO_2_ at 37 °C for 24 h. CCK-8 (10 µL/well) was then added to 100 µL aliquots from each cell and incubated 2–4 h. Absorbances were measured at 450 nm using a microplate reader (Versamax, Molecular Devices, San Jose, CA, USA).

### Docking simulation of ellagic acid derivatives with AChE and MAO-B

To simulate dockings of **3**, **4**, and **5** with AChE and MAO-B, we used AutoDock Vina^30^, which has an automated docking facility. To define enzyme binding pockets, we used active sites defined by a complex of AChE with 4-carbamoyl-1-(3-{2-[(*E*)-(hydroxyimino)methyl]-1*H*-imidazol-1-yl}propyl)pyridin-1-ium (LND) (PDB ID: 6O5V) and a complex of MAO-B with (R)-rosiglitazone (RGZ) (PDB ID: 4A7A). To prepare **3**, **4**, and **5** for docking simulations, we performed the following steps: (1) created 2D structures of the three compounds, (2) converted these 2D structures into 3D structures, and (3) performed energy minimization using the ChemOffice program (http://www.cambridgesoft.com). Docking simulations of AChE or MAO-B with **3**, **4**, and **5** were performed using AutoDock Vina 1.1.2^31^. Based on the docking results, we checked for possible hydrogen bonding interactions with bonding relaxation constraints of 0.4 Å and 10.0° using Chimera 1.15 program^32^.

### Pharmacokinetics by SwissADME

Pharmacokinetic analyses for drug-like properties were performed in silico on **3**, **4**, and **5** using the SwissADME web tool at http://www.swissadme.ch/^33^.

## Results

### Isolation and identification of compounds in *C. cuspidata* extract by bioassay-guided fractionation using AChE inhibitory activity

**Figure 1:**
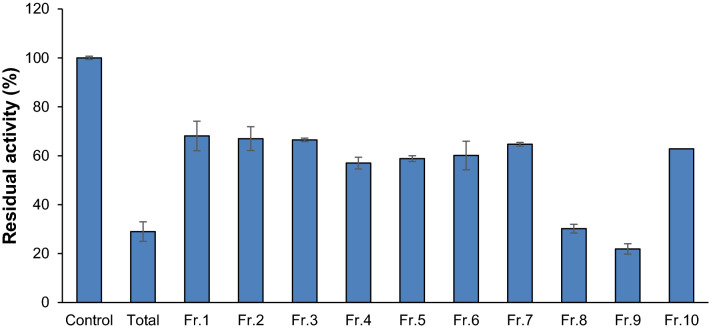
AChE inhibitory activities of the methanol extract of *C. cuspidata* and the fractions obtained by the preparative HPLC. Residual activities were measured at extract and fraction concentrations of 50 µg/mL.

Of the 276 herbal extracts tested, the methanol extract of *C. cuspidata* stems was selected based on its AChE inhibitory activity, novelty of the plant, and availability of raw material. During preparative HPLC of the extract, eluents were divided into ten fractions. AChE inhibitory analysis showed that fractions 8 (CCS-8) and 9 (CCS-9) had the lowest residual activities of 30.2 and 21.9%, respectively, at 50 µg/mL (Fig. 1).

**Figure 2:**
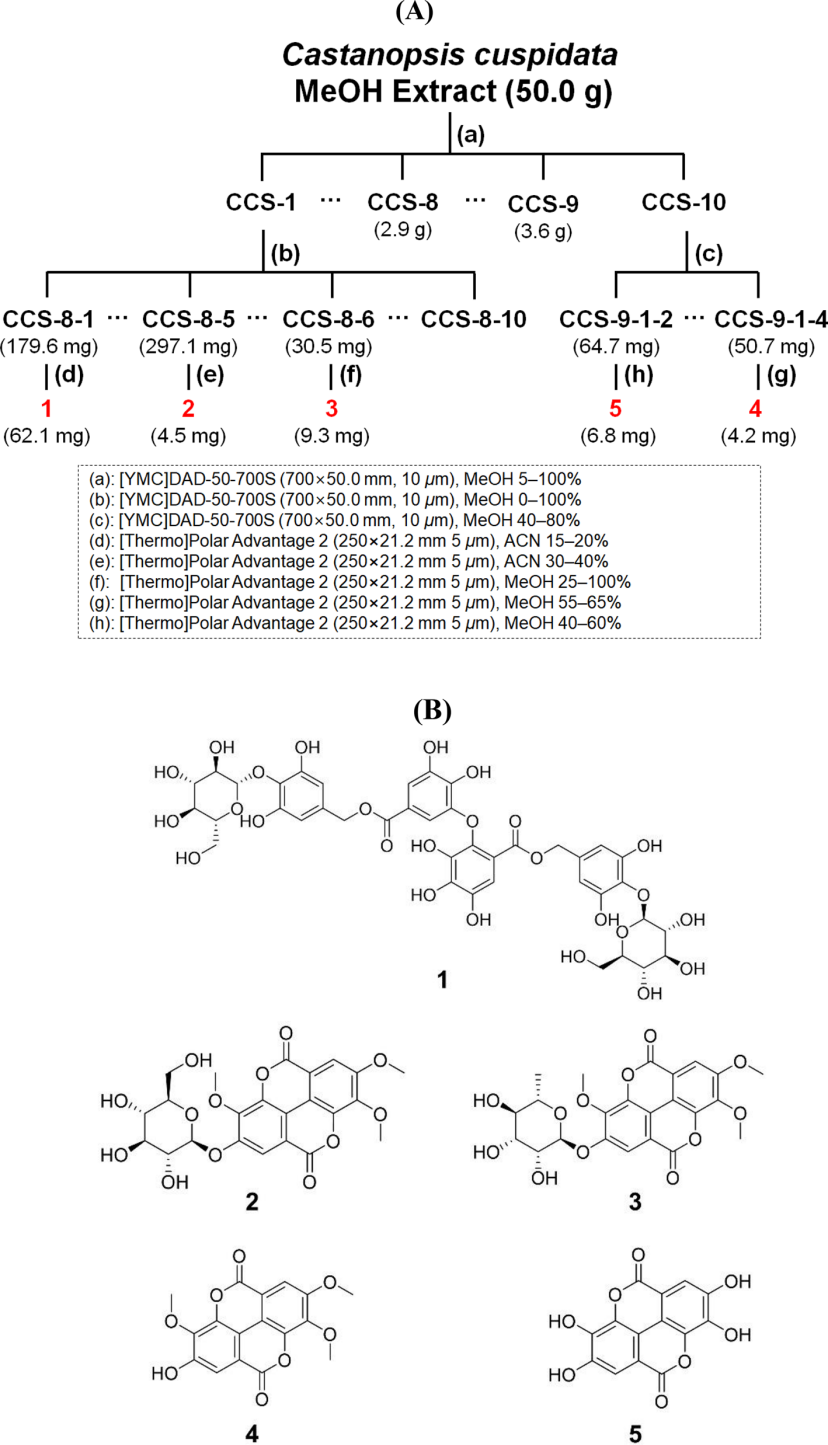
Scheme for the isolation of compounds **1**–**5** from *C. cuspidata* (**A**) and their chemical structures (**B**); chestanin (**1**), 4′-*O-*(*β*-d-glucopyranosyl)-3,3′,4-tri-*O*-methylellagic acid (**2**), 4′-*O-*(*α*-l-rhamnopyranosyl)-3,3′,4-tri-*O*-methylellagic acid (**3**), 3,3′,4-tri-*O*-methylellagic acid (**4**), and ellagic acid (**5**).

Compounds **1**–**5** were isolated from CCS-8 and CCS-9 by bioactivity-guided fractionation. The chemical structures of **1**–**5** were determined by comparing spectroscopic data, that is, ^1^H, ^13^C, 2D NMR (COSY, HMQC, and HMBC), and HRESIMS data with literature values. ^1^H, ^13^C, and MS data are provided in Supplementary Materials (Figs. S1–S10). Compounds **1**–**5** were identified as chestanin (**1**)^34^, 4′-*O*-(*β*-d-glucopyranosyl)-3,3′,4-tri-*O*-methylellagic acid (**2**)^35^, 4′-*O*-(*α*-l-rhamnopyranosyl)-3,3′,4-tri-*O*-methylellagic acid (**3**)^36^, 3,3′,4-tri-*O*-methylellagic acid (**4**)^37^, and ellagic acid (**5**)^37^. The purities of **1**–**5** were 95.2%, 99.1%, 99.1%, 92.0%, and 95.1% as determined by UPLC-PDA analysis (Fig. S11). A scheme for the purification process is provided in Fig. 2A, and the structures of the five isolated compounds are provided in Fig. 2B.

Chestanin (**1**) was previously isolated from the leaves of *C. cuspidata* and its structure elucidated, however, compound **5**, that was ellagic acid, and compounds **2**–**4**, which were identified as ellagic acid derivatives, were first isolated from *C. cuspidata* in this study. Ellagic acid, and ellagic acid derivatives may be considered secondary metabolites of the biosynthesis of gallic acid^38^.

### Inhibitory activities of the five isolated compounds

The five compounds isolated (named **1** to **5**) were assayed for inhibitory activities against five enzymes, namely, AChE, BChE, MAO-A, MAO-B, and BACE-1. **3** and **4** inhibited AChE by about 50% at 10 µM, whereas the other three compounds inhibited it by less than 30% (Table 1). **3** and **5** inhibited MAO-B by more than 50%. All five compounds weakly inhibited BChE, MAO-A, and BACE-1. Regarding IC_50_ values, **3** and **4** had similar IC_50_ values of 10.1 and 10.7 µM, respectively, for AChE (Table 1), and had moderate selectivity index (SI) values of 3.96 and 3.73, respectively, for AChE over BChE. In addition, **3** and **5** had IC_50_ values of 7.27 and 9.21 µM, respectively, for MAO-B. Collectively, these results suggest that **3** is a dual-acting inhibitor for AChE and MAO-B and that **4** and **5** are effective AChE and MAO-B inhibitors, respectively.

**Table 1:** Inhibitions of AChE, BChE, MAO-A, MAO-B, and BACE-1 by the compounds isolated from *C. cuspidata*^a^.

Compounds	Residual activity at 10 µM (%)					IC_50_ (µM)				SI^b^
	AChE	BChE	MAO-A	MAO-B	BACE-1	AChE	BChE	MAO-A	MAO-B	
**1**	72.5 ± 2.1	96.2 ± 3.9	91.7 ± 1.6	67.2 ± 8.1	78.9 ± 0.3	37.0 ± 3.0	> 40	> 40	> 40	> 1.1
**2**	70.5 ± 5.0	90.8 ± 4.2	91.3 ± 7.9	60.9 ± 5.2	91.9 ± 1.0	46.4 ± 2.7	> 40	> 40	> 40	> 0.9
**3**	55.4 ± 3.4	83.3 ± 2.1	83.0 ± 6.3	43.2 ± 3.7	99.7 ± 0.4	10.1 ± 0.4	> 40	> 40	7.27 ± 0.16	> 4.0
**4**	53.2 ± 0.0	92.3 ± 2.3	83.1 ± 4.7	89.1 ± 4.1	90.1 ± 0.4	10.7 ± 1.0	> 40	> 40	> 40	> 3.7
**5**	81.5 ± 4.2	96.9 ± 5.8	99.5 ± 0.7	45.4 ± 4.1	59.1 ± 0.3	41.7 ± 2.4	> 40	> 40	9.21 ± 0.16	> 1.0
Toloxatone								1.08 ± 0.03	-	
Lazabemide								–	0.14 ± 0.01	
Clorgyline								0.0070 ± 0.0007	–	
Pargyline								–	0.030 ± 0.001	
Tacrine						0.27 ± 0.02	0.060 ± 0.002			
Donepezil						0.0095 ± 0.0019	0.180 ± 0.004			

^a^Results shown are the means ± SDs of duplicate or triplicate experiments.

^b^SI values were calculated by dividing the IC_50_ for BChE by IC_50_ for AChE.

### Reversibility analyses of AChE or MAO-B inhibitions by 3, 4, or 5

**Figure 3:**
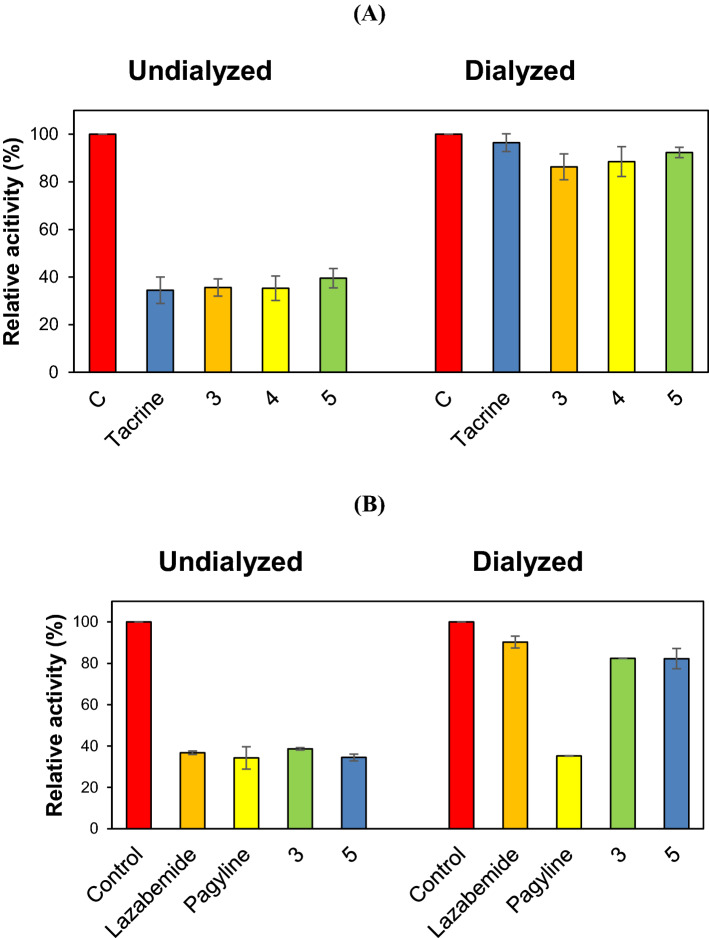
Dialysis recoveries of AChE inhibitions by **3**, **4**, and **5** (**A**), and of MAO-B inhibitions by **3** and **5** (**B**). The concentrations of **3**, **4**, **5**, and tacrine used were 25, 20, 80, and 0.60 µM, respectively, and the concentrations of **3**, **5**, lazabemide, and pargyline used were 14, 20, 0.28, and 0.06 µM, respectively. Tacrine was used as a reference reversible AChE inhibitor, and lazabemide and pargyline were used as reference reversible and irreversible MAO-B inhibitors, respectively. After preincubating the compounds with enzymes for 15 min, the mixtures were dialyzed for 6 h with two buffer changes. Results are the averages of duplicate or triplicate experiments.

Reversibilities of AChE inhibitions by **3**, **4**, or **5** were investigated by dialysis. Inhibitions of AChE by **3**, **4**, and **5** were substantially recovered from 35.6% (A_U_) to 86.3% (A_D_), from 35.3% to 88.5%, and from 35.3% to 92.3%, respectively, and these values were similar to those observed for the reversible inhibitor tacrine (34.5 to 96.5%) (Fig. 3A). Inhibitions of MAO-B by **3** and **5** were markedly recovered by dialysis from 38.7 to 82.4%, from 34.5 to 82.3%, respectively, and these values were similar to those observed of the reversible inhibitor lazabemide (36.8 to 90.3%), but not to those of the irreversible inhibitor pargyline (34.3 to 35.2%) (Fig. 3B). These results indicate that **3**, **4**, and **5** are reversible inhibitors of AChE, and that **3** and **5** are reversible inhibitors of MAO-B.

### Kinetics of AChE or MAO-B inhibitions by 3, 4, or 5

**Figure 4:**
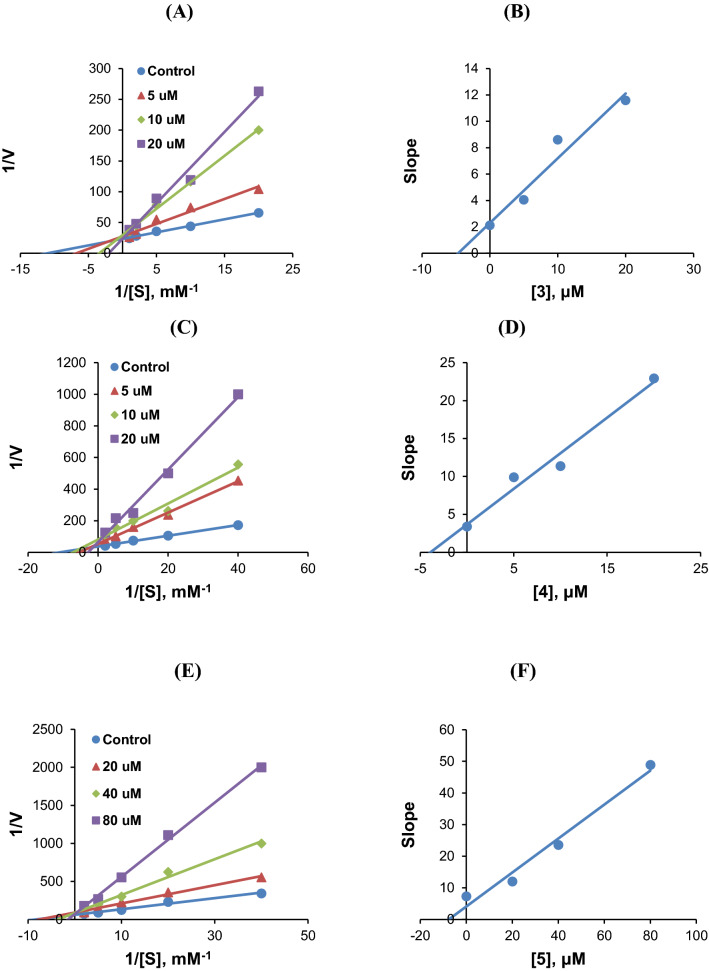
Lineweaver–Burk (LB) plots of AChE inhibitions by **3**, **4**, and **5** (**A**, **C**, and **E**, respectively), and their respective secondary plots (**B**, **D**, and **F**, respectively) of the slopes of LB plots versus inhibitor concentrations. Substrate concentrations ranged from 0.05 to 1.0 mM. Experiments were carried out at three inhibitor concentrations, i.e., at ~ 0.5 × , 1.0 × , and 2.0 × IC_50_ values. Initial reaction rates are expressed as increases in absorbance per min.

Modes of AChE inhibitions by **3**, **4**, or **5** were investigated using LB plots. According to these plots, AChE inhibitions by **3**, **4**, or **5** were competitive but partly mixed (Fig. 4A,C,E, respectively). Secondary plots of the slopes of LB plots against inhibitor concentrations showed that the K_i_ values of **3**, **4**, and **5** were 5.37 ± 0.62, 3.74 ± 0.26, and 9.43 ± 2.51 µM (Figs. 4B,D,F, respectively). These results indicate that compounds **3**, **4**, and **5** acted as competitive but partly mixed inhibitors of AChE.

**Figure 5:**
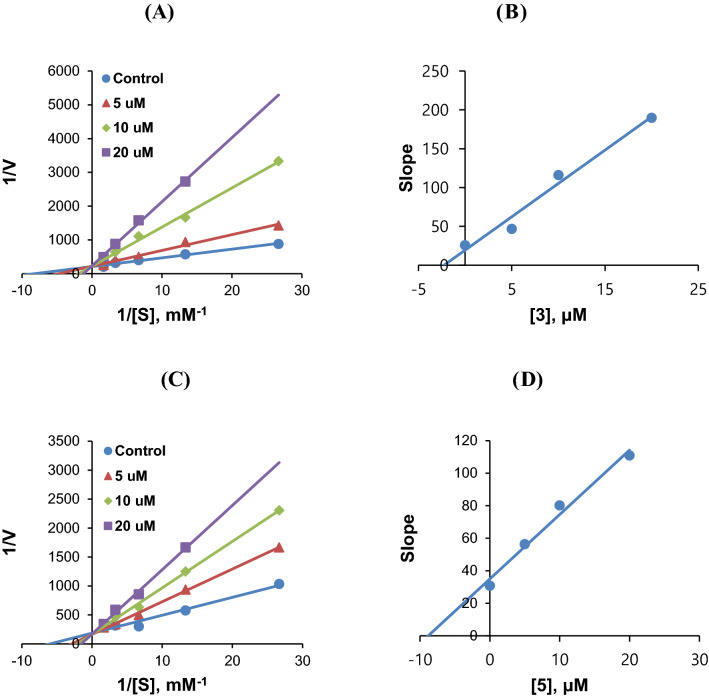
LB plots of MAO-B inhibitions by **3** (**A**) and **5** (**C**), and their secondary plots (**B** and **D**, respectively) of LB slopes vs. inhibitor concentrations. Substrate concentrations ranged from 0.0375 to 0.6 mM. Experiments were carried out at three inhibitor concentrations, i.e., at ~ 0.5 × , 1.0 × , and 2.0 × IC_50_ values.

Regarding the inhibition of MAO-B, **3** and **5** were competitive inhibitors (Fig. 5A,C), and secondary plots showed that their K_i_ values of **3** and **5** were 2.25 ± 0.01 and 7.51 ± 1.86 µM, respectively (Fig. 5B,D). These results suggest that **3** and **5** are competitive inhibitors of MAO-B.

### Cytotoxicities of 3, 4, and 5

**Figure 6:**
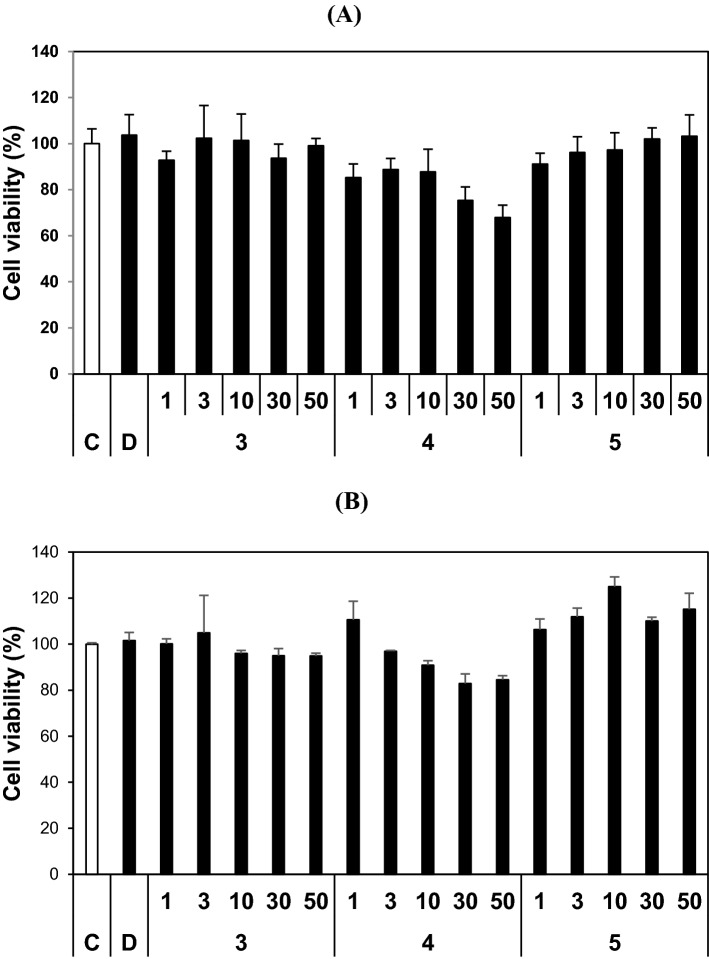
Effects of **3**, **4**, and **5** on the viabilities of MDCK (**A**) and HL-60 (**B**) cells. Both cell lines were treated with each compound (at 1, 3, 10, 30, or 50 µM) for 24 h. Culture supernatants were then removed and CCK-8 was added. C, control without compounds; D, control treated with 0.1% DMSO. Data are expressed as the means ± SDs of triplicate experiments.

The effects of **3**, **4**, and **5** on the viabilities of MDCK and HL-60 cells were investigated using the CCK-8 assay. **3** and **5** showed negligible effects on the viabilities of MDCK (normal cell line) or HL-60 (cancer cell line) cells at 50 µM (Fig. 6). However, at this concentration, **4** reduced MDCK and HL-60 viabilities to 67.9% and 84.6%, respectively. These results suggest that **3** and **5** are non-toxic to the normal and cancer cells and that **4** is slightly toxic to both cell types.

### Molecular docking simulations of 3, 4, or 5 with AChE or MAO-B

**Figure 7:**
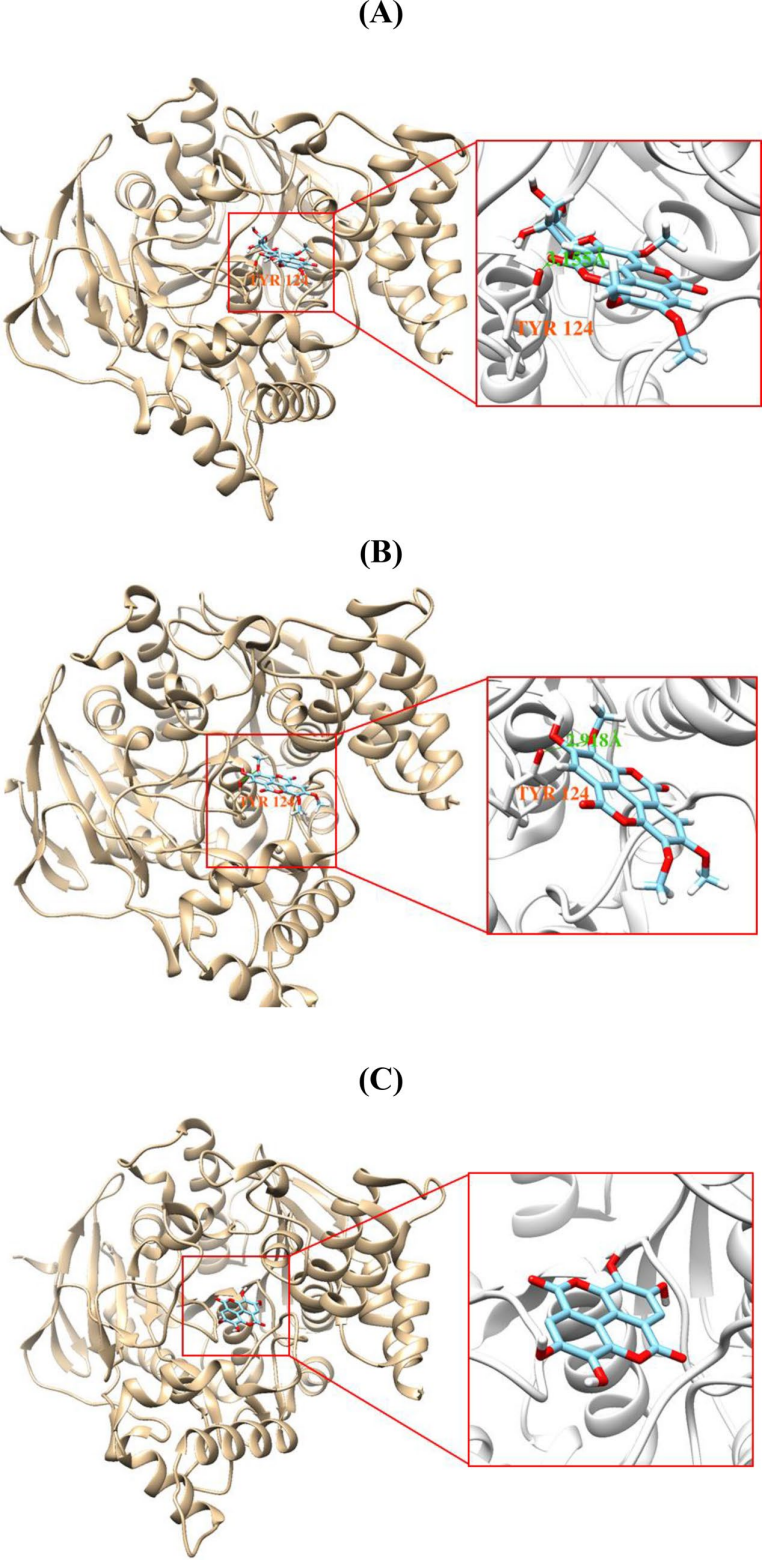
Docking simulations of **3** (**A**), **4** (**B**), and **5** (**C**) with AChE (6O5V). **3** and **4** formed a single hydrogen bond with Try124 of AChE at distances of 3.155 and 2.918 Å, respectively. **3**, 4′-*O*-(α-l-rhamnopyranosyl)-3,3′,4-tri-*O*-methylellagicacid; **4**, 3,3′,4-tri-*O*-methyl-ellagicacid; **5**, ellagic acid. Docking simulations were performed using AutoDock Vina 1.1.2. In addition, the structures were visualized by Chimera 1.15 program.

**Figure 8:**
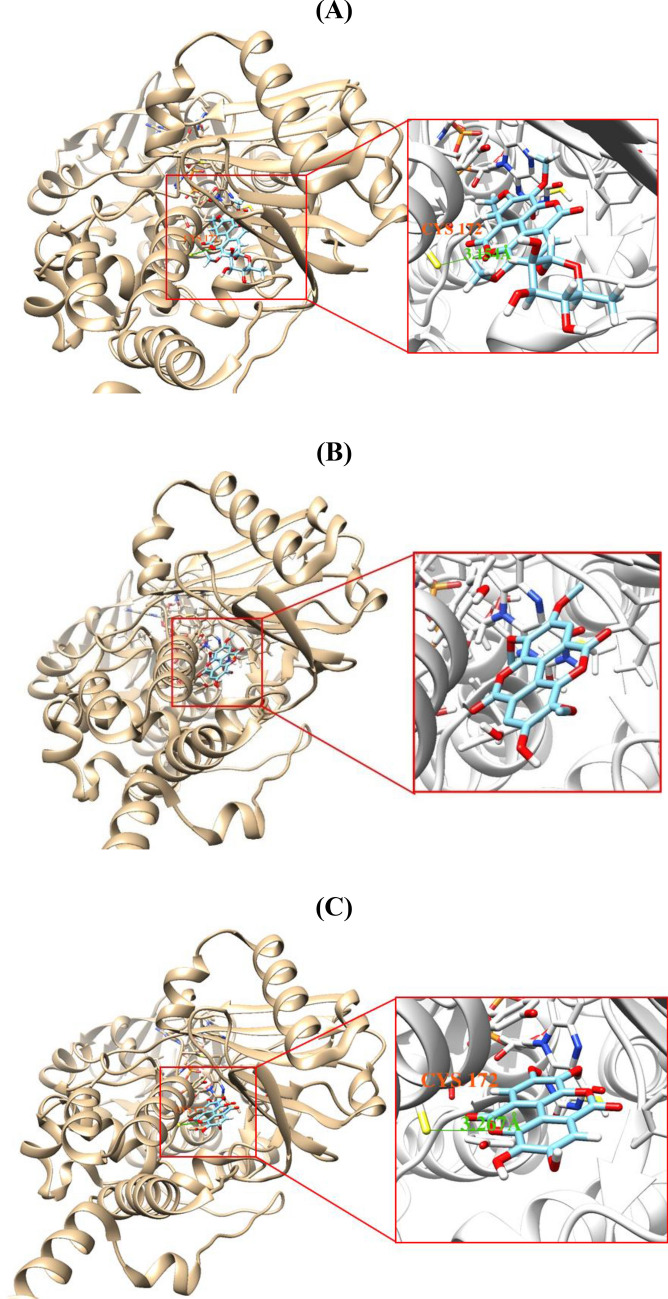
Docking simulations of **3** (**A**), **4** (**B**), and** 5** (**C**) with MAO-B (4A7A). **3** and **5** both formed a single hydrogen bond interaction with Cys172 of MAO-B at distances of 3.154 and 3.267 Å, respectively. Docking simulations were performed using AutoDock Vina 1.1.2. In addition, the structures were visualized by Chimera 1.15 program.

Docking simulations showed that **3**, **4**, and **5** were located properly at the binding sites of LND complexed with AChE and of RGZ complexed with MAO-B. AutoDock Vina predicted that the binding energies of **3**, **4**, and **5** to AChE were − 8.5, − 9.2, and − 8.3 kcal/mol, respectively, and those to MAO-B were − 7.3, − 4.7, − 8.9 kcal/mol, respectively (Table 2). Docking simulation results showed that **3** or **4** formed a hydrogen bond with Tyr124 of AChE at distances of 3.155 and 2.918 Å, respectively, but that **5** did not form a hydrogen bond with AChE (Fig. 7). On the other hand, **3** or **5** formed a hydrogen-bond with Cys172 of MAO-B at distances of 3.154 and 3.267 Å, respectively. **4** was not predicted to form a hydrogen bond with MAO-B (Fig. 8).

**Table 2:** Docking scores and predicted hydrogen bond(s) of the three compounds with AChE or MAO-B^*^

Compounds	Docking scores (kcal/mol)		Hydrogen bond(s) predicted	
	AChE	MAO-B	AChE	MAO-B
**3**	− 8.5	− 7.3	Tyr124	Cys172
**4**	− 9.2	− 4.7	Tyr124	
**5**	− 8.3	− 8.9		Cys172

* Determined by AutoDock Vina.

### In silico pharmacokinetics of 3, 4, and 5 as determined by SwissADME

In silico pharmacokinetic studies predicted the gastrointestinal (GI) absorptions of **4** and **5** are high, but that of **3** is low (Table 3). The compounds were not found to be P-glycoprotein (P-gp) substrates and not capable of crossing the blood–brain barrier (BBB). **3** was predicted to have the highest skin permeability (-8.74 cm/s).

**Table 3:** Predicted pharmacokinetic properties of **3**, **4**, and **5** by SwissADME.

Compound	GI absorption	BBB permeant	P-gp substrate	CYP1A2 inhibitor	CYP2C19 inhibitor	CYP2C9 inhibitor	CYP2D6 inhibitor	CYP3A4 inhibitor	Log *K*_*p*_ (skin permeation) (cm/s)
**3**	Low	No	No	No	No	No	No	No	− 8.74
**4**	High	No	No	Yes	No	Yes	No	Yes	− 6.92
**5**	High	No	No	Yes	No	No	No	No	− 7.36

GI: gastrointestinal; BBB: blood–brain barrier; P-gp: P-glycoprotein; CYP: Cytochrome P450.

## Discussion

In our previous studies, we isolated the MAO-A inhibitors, hispidol from *Glycin max* Merrill^39^ and decursin from *Angelica gigas* Nakai^40^, and the MAO-B inhibitors, maackiain from *Sophora flavescens*^21^ and calycosin from *Maackia amurensis*^11^. In the present study, of the 276 herbal extracts examined, the methanol extract of the stems of *C. cuspidata* was selected as a potential source of AChE inhibitors and five compounds were subsequently isolated using a bioassay-guided method and identified.

Of the five compounds isolated, **3** and **4** effectively inhibited AChE, and **3** and **5** effectively inhibited MAO-B. Our results suggest that **3** is a dual-acting inhibitor of AChE and MAO-B, **4** is an AChE inhibitor, and **5** is a MAO-B inhibitor. Little information is available on natural dual AChE/MAO-B inhibitors, though, recently, macelignan was identified as a dual AChE/MAO-B inhibitor with IC_50_ values of 4.16 and 7.42 µM, respectively^22^.

Ellagic acid, **5**, is a natural polyphenol with anti-proliferative, anti-oxidant, anti-diabetic, anticancer, and apoptosis-inducing activities^41,42^. Recently, ellagic acid was reported to have neuroprotective and cognition-enhancing effects in sporadic AD based on behavioral investigations, however, its IC_50_ for AChE was 132.92 μM, which was 3.2 times higher than our result (IC_50_ = 41.7 μM)^43^. Furthermore, the previously reported IC_50_ of ellagic acid for MAO-B was 0.412 μM using rat brain mitochondrial fractions, which was much lower than our result (IC_50_ = 9.21 μM)^44^. The inhibitory activities of the ellagic acid derivatives **3** and **4** on AChE and MAO-B have not been previously described.

Natural AChE inhibitors have been classified into three groups according to their IC_50_ values, i.e., high potency, IC_50_ < 15 μM; moderate potency, 15 < IC_50_ < 50 μM; and low potency, 50 < IC_50_ < 1000 μM^45^. Based on this classification, **3** (IC_50_ = 10.1 µM) and **4** (IC_50_ = 10.7 µM) are highly potent AChE inhibitors. The potencies of **3** and **4** were ~ 5 times lower or ~ 2 times higher than that of galantamine [IC_50_ values = 2.16 µM^46^ or 21.1 µM (6.07 µg/ml)^47^], which is a natural compound and used for the treatment of AD. Polyphenols that target AChE have attracted research interest as potential therapeutics for AD^48^. The AChE inhibitory potencies of **3** and **4** were higher than those previously reported for other natural polyphenol, e.g., *C*-glucosylflavone, isovitexin-7-*O*-methyl ether (swertisin) (IC_50_ = 32.09 µg/mL, i.e., 71.9 µM) from *Anthocleista vogelii*^49^, the flavonoids tiliroside (IC_50_ = 23.5 µM) and quercetin (IC_50_ = 19.8 µM) from *Agrimonia pilosa*^50^, curcumin (IC_50_ = 23.5 µM) from *Curcuma longa*^51^, and epigallocatechin gallate from green tea (IC_50_ = 14.8 µM)^52^, but lower than that of a xanthonoid α-mangostin (IC_50_ = 2.48^53^ or 6.3 µM^54^), a hydroxycinnamoylated catechin (IC_50_ = 2.49 µM) from *Camellia sinensis* var. *assamica*^55^, and two resveratrol oligomers vitisin A (IC_50_ = 1.04 µM) and heyneanol A (IC_50_ = 1.66 µM) from *Vitis amurensis*^56^. The potencies of **3** and **4** were similar to those of two khellactone derivatives (IC_50_ = 9.28 and 10.0 μM) from *Peucedanum japonicum* Thunberg^26^.

**3**, **4**, and **5** inhibited AChE in a competitive but partially mixed manner, whereas **3** and **5** inhibited MAO-B in a competitive manner. The majority of AChE inhibitors described to date are mixed or partially mixed inhibitors^22,26,57^, whereas the MAO-B inhibitors described are usually competitive type^58–60^.

Regarding structure–activity relationships (SARs), the 3,3′,4-tri-*O*-methyl group of **4** (IC_50_ = 10.7 μM) and the α-L-rhamnopyranosyl-3,3′,4-tri-*O*-methyl groups of **3** (IC_50_ = 10.1 μM) increased AChE inhibitory activity as compared with the **5** parent (IC_50_ = 41.7 μM). However, the β-D-glucopyranosyl group of **2** (IC_50_ = 46.4 μM), which replaced the α-L-rhamnopyranosyl group of **3** decreased AChE inhibitory activity to the level of **5**. As regards MAO-B inhibitory activities, the α-L-rhamnopyranosyl of **3** (IC_50_ = 7.27 μM) and **5** (IC_50_ = 9.21 μM) increased the activity as compared with the 3,3′,4-tri-*O*-methyl group of **4** and the β-d-glucopyranosyl group of **2** (IC_50_ values > 40 μM).

Docking analysis showed that the binding energies of **3** (− 8.5 kcal/mol) and **4** (− 9.2 kcal/mol) for AChE were higher than that of **5** (− 8.3 kcal/mol), largely due to hydrogen bond formation, which was not predicted for **5**, and these results concur with their IC_50_ values as determined by inhibition assays, (i.e., 10.1, 10.7, and 41.7 μM of **3**, **4**, and **5**, respectively). Regarding docking to MAO-B, **3** and **5** both formed a hydrogen bond, whereas **4** did not, and the binding energies of **3** (− 7.3 kcal/mol) and **5** (− 8.9 kcal/mol) for MAO-B were higher than that of **4** (− 4.7 kcal/mol), in line with their IC50 values (i.e., 7.27, > 40, and 9.21 μM of **3**, **4**, and **5**, respectively). We attribute these differences to a combination of hydrogen bond formation, electrostatic bonding, van der Waals forces, dissolvent effects^61^, and the structural flexibilities of the compounds as determined by AutoDock program^62^.

Our in silico pharmacokinetic studies also predicted that **3**, **4** and **5** are absorbed well in the GI tract, are not substrates for P-gp, which causes drug efflux to gut lumen, and inhibit some cytochrome P450, but do not cross the BBB. In terms of cytotoxicity, **3**, **4**, and **5** were non-toxic or slightly toxic to the normal and cancer cells at 50 μM. Ellagic acid and its derivatives are able to react with a polycyclic aromatic hydrocarbon metabolite, the ultimate carcinogen, and prevent its covalent binding to DNA^63^. However, some of them exhibited DNA-damaging activity in DNA repair-deficient yeast^64^. Therefore, when compounds **3**, **4**, and **5** are used, careful doses should be needed, though the compounds were non- or slightly toxic.

In summary, we isolated the highly potent, reversible, selective AChE inhibitors **3** and **4** from *Castanopsis cuspidata* var. *sieboldii*, and show that these compounds have potential use for the treatment of neurological diseases like AD.

## Conclusion

Five compounds were isolated from the methanol extract of the stems of *Castanopsis cuspidata* var. *sieboldii* by activity-guided screening for AChE inhibitors. Of these, **3** and **4** effectively and selectively inhibited AChE (IC_50_ = 10.1 and 10.7 µM, respectively). In addition, **3** effectively inhibited monoamine MAO-B (IC_50_ = 7.27 µM) followed by **5** (IC_50_ = 9.21 µM). Binding energies of **3** and **4** (− 8.5 and − 9.2 kcal/mol, respectively) for AChE were higher than that of **5** (− 8.3 kcal/mol) due to hydrogen bonding. In addition, compounds 3, 4, and 5 were slightly or non-toxic to MDCK cells. All five compounds weakly inhibited BChE and BACE-1. Our results show that **3** is a dual-targeting AChE and MAO-B inhibitor, **4** is an AChE inhibitor, and **5** is a MAO-B inhibitor, and suggest the potential use of these compounds for the treatment of AD.

## Supplementary Information

Supplementary Figures.
