# Supplementary material for: Acetylcholinesterase and monoamine oxidase-B inhibitory activities by ellagic acid derivatives isolated from Castanopsis cuspidata var. sieboldii
Source: Sci Rep. 2021 Jul 6;11:13953. doi: 10.1038/s41598-021-93458-4 (PMC8260592; doi:10.1038/s41598-021-93458-4)
Supplement: Supplementary file 1 — Supplementary Figures. [file 41598_2021_93458_MOESM1_ESM.docx]

**Supplementary Materials**

**Table of Contents**

**Figure S1.** ^1^H and ^13^C NMR (500 and 125 MHz, methanol-*d*_4_) spectra of chestanin (**1**) 2

**Figure S2.** HR-ESI-MS spectrum of chestanin (**1**). 3

**Figure S3.** ^1^H and ^13^C NMR (600 and 150 MHz, DMSO-*d*_6_) spectra of 4'-*O-*(*β*-d-glucopyranosyl)-3,3',4-tri-*O*-methylellagic acid (**2**). 4

**Figure S4.** HR-ESI-MS spectrum of 4'-*O-*(*β*-d-glucopyranosyl)-3,3',4-tri-*O*-methylellagic acid (**2**). 5

**Figure S5.** ^1^H and ^13^C NMR (600 and 150 MHz, DMSO-*d_6_*) spectra of 4'-*O-*(*α*-l-rhamnopyranosyl)-3,3',4-tri-*O*-methylellagic acid (**3**). 6

**Figure S6.** HR-ESI-MS spectra of 4'-*O-*(*α*-l-rhamnopyranosyl)-3,3',4-tri-*O*-methylellagic acid (**3**). 7

**Figure S7.** ^1^H and ^13^C NMR (500 and 100 MHz, DMSO-*d_6_*) spectra of 3,3',4-tri-*O*-methylellagic acid (**4**). 8

**Figure S8.** HR-ESI-MS spectra of 3,3',4-tri-*O*-methylellagic acid (**4**). 9

**Figure S9.** ^1^H and ^13^C NMR (400 and 100 MHz, DMSO-*d_6_*) spectra of ellagic acid (**5**). 10

**Figure S10.** HR-ESI-MS spectrum of ellagic acid (**5**). 11

**Figure S11.** The purities of the isolated compounds **1**–**5**. 12


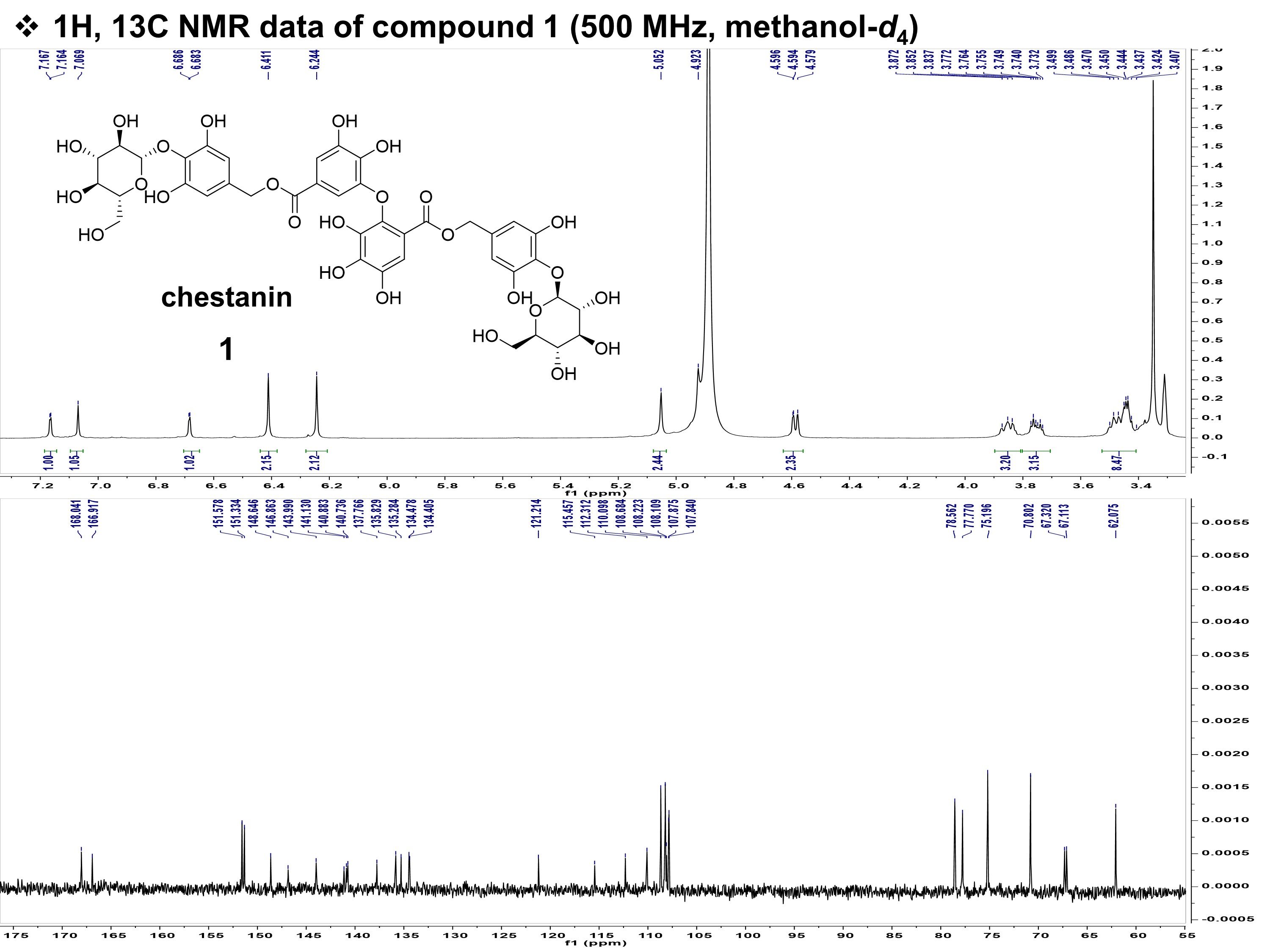


**Figure S1.** ^1^H and ^13^C NMR (500 and 125 MHz, methanol-*d*_4_) spectra of chestanin (**1**).


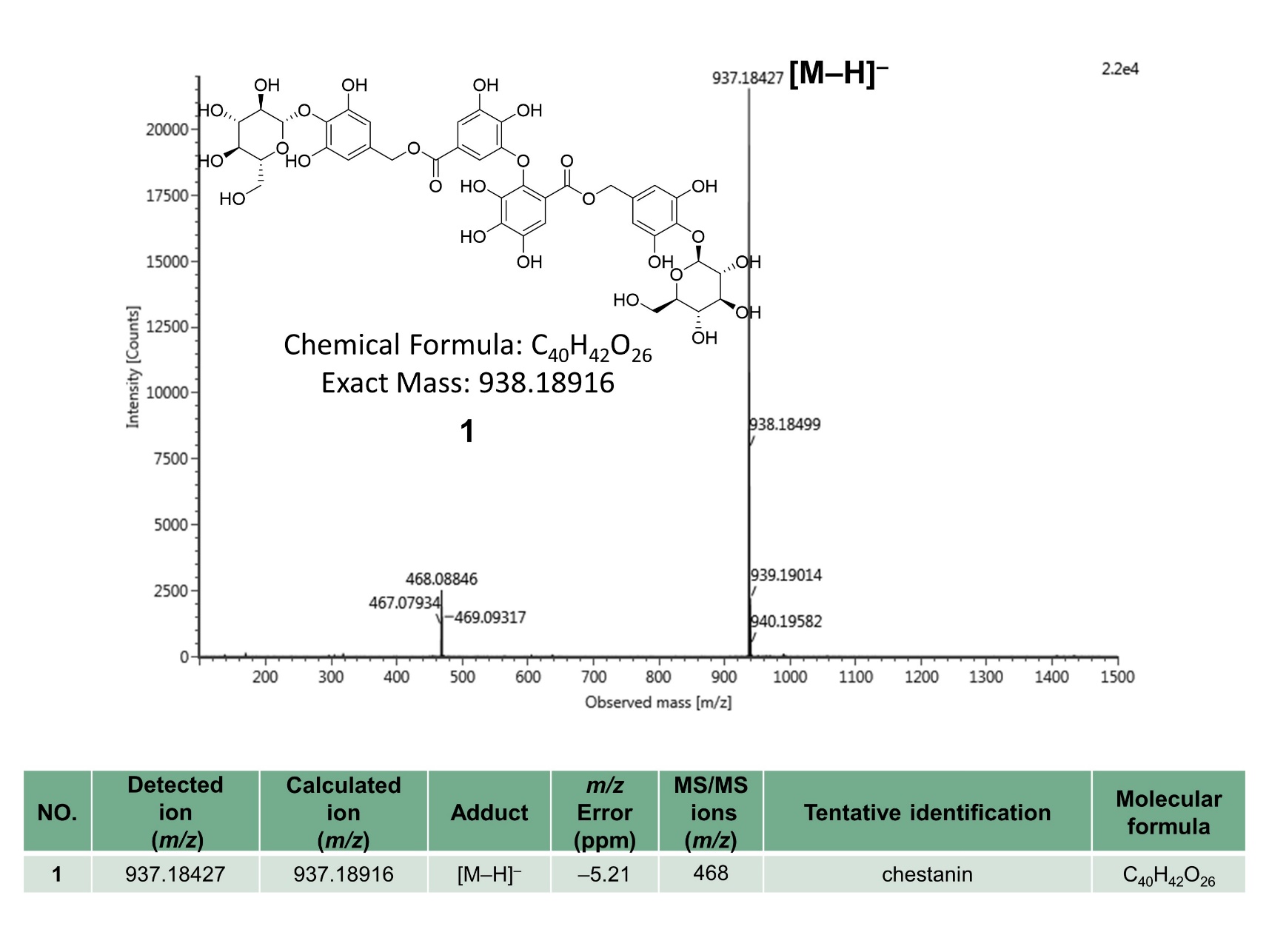


**Figure S2.** HR-ESI-MS spectrum of chestanin (**1**).


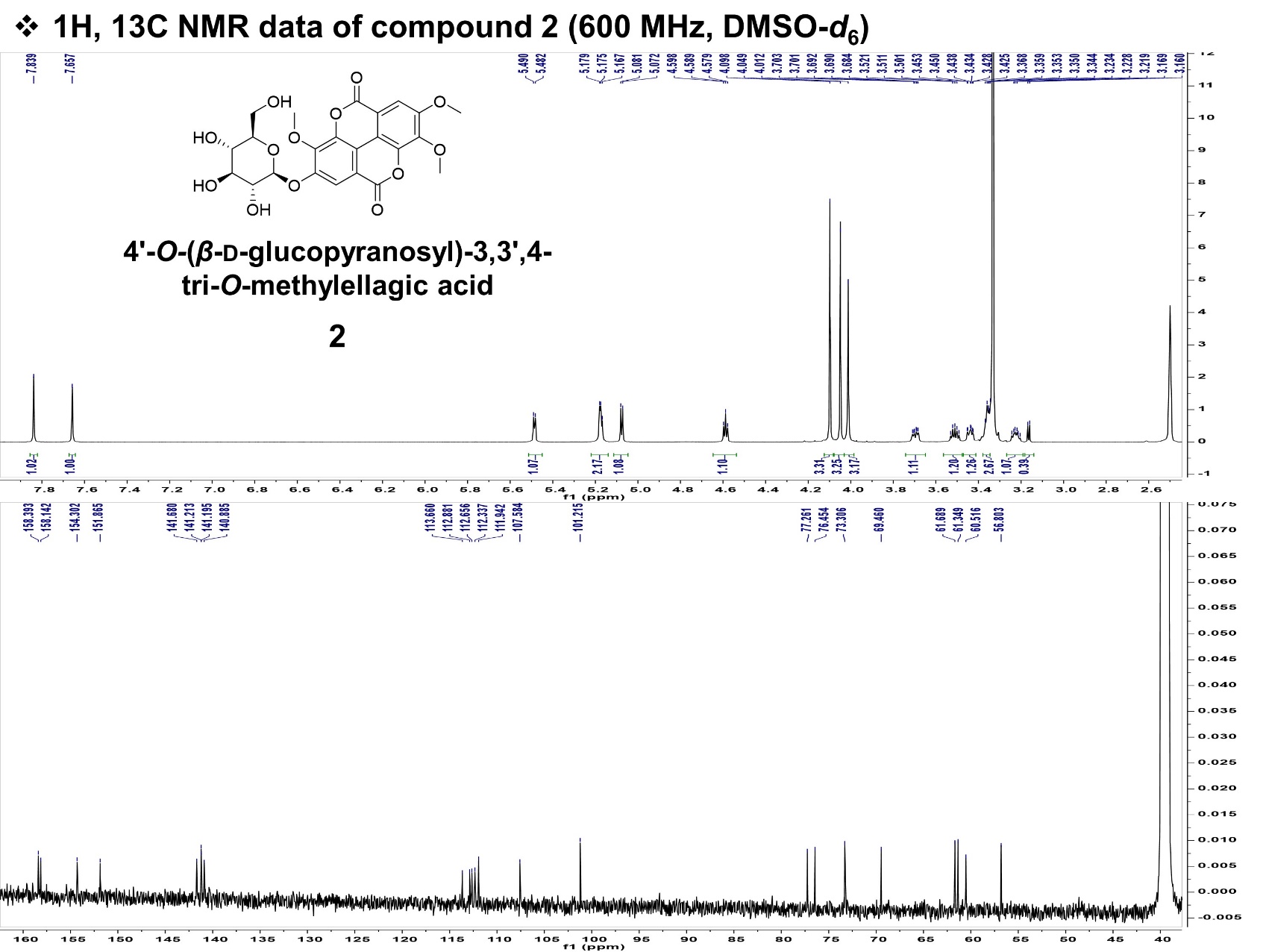


**Figure S3.** ^1^H and ^13^C NMR (600 and 150 MHz, DMSO-*d*_6_) spectra of 4'-*O-*(*β*-d-glucopyranosyl)-3,3',4-tri-*O*-methylellagic acid (**2**).


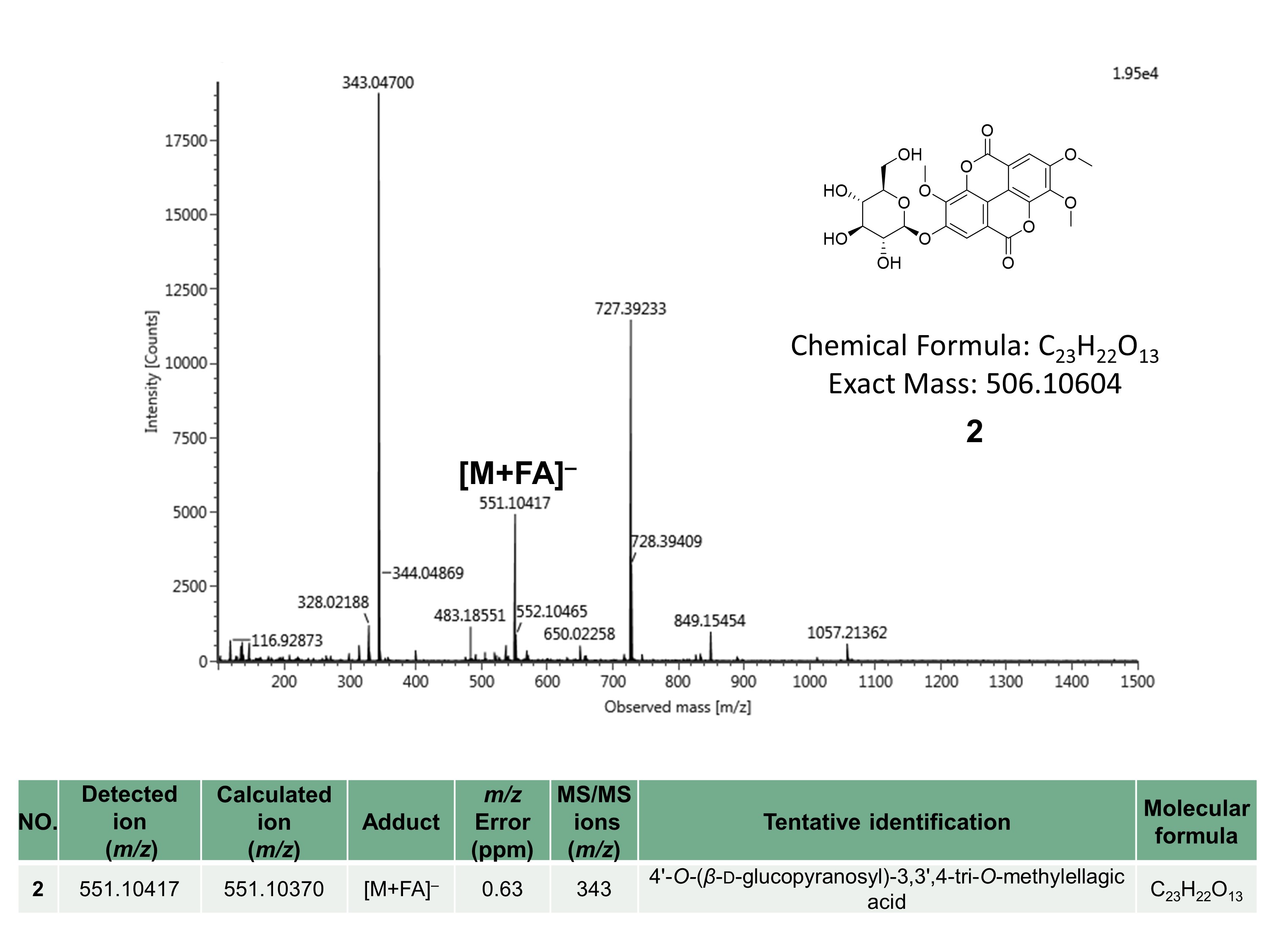


**Figure S4.** HR-ESI-MS spectrum of 4'-*O-*(*β*-d-glucopyranosyl)-3,3',4-tri-*O*-methylellagic acid (**2**).


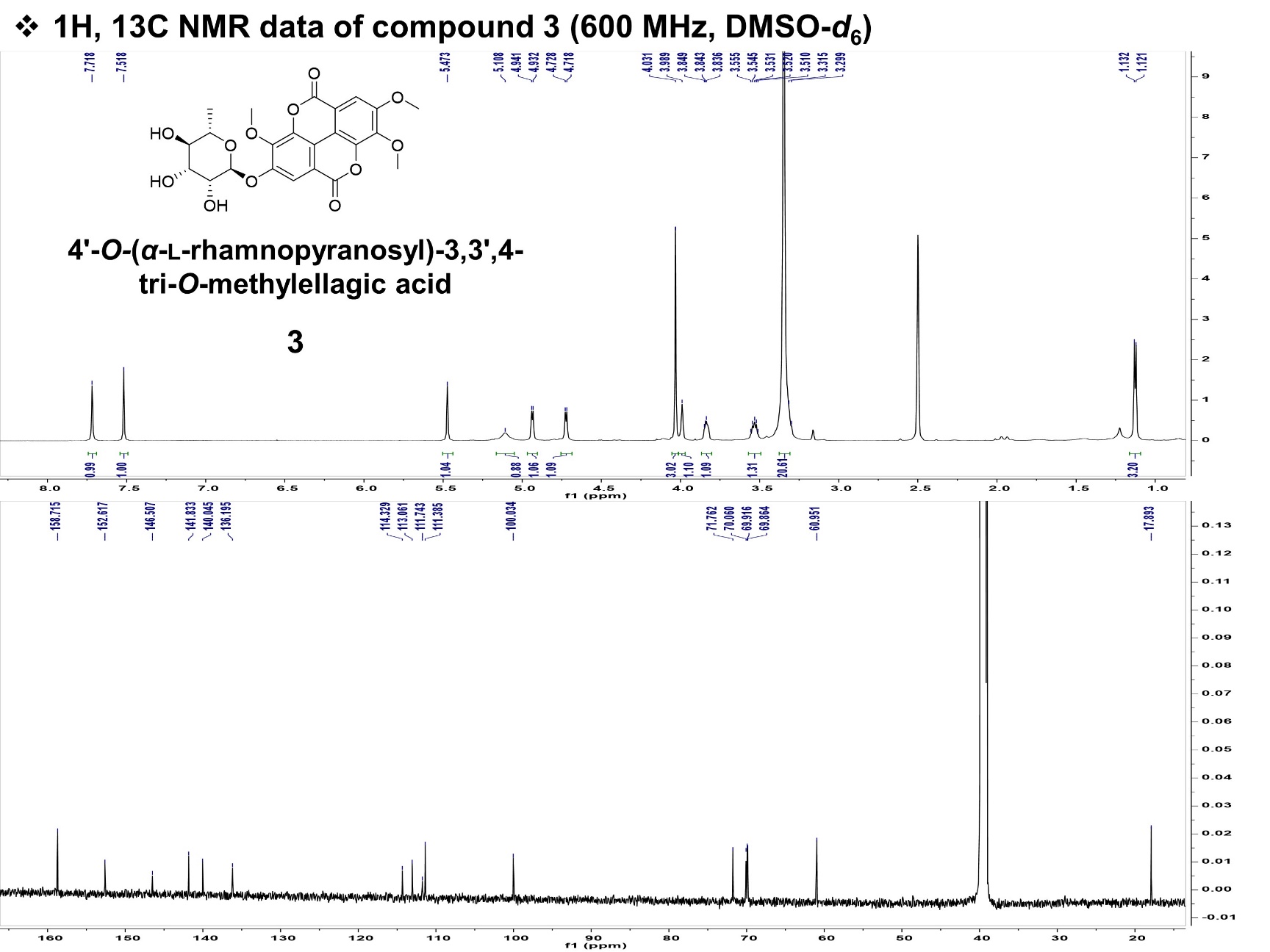


**Figure S5.** ^1^H and ^13^C NMR (600 and 150 MHz, DMSO-*d_6_*) spectra of 4'-*O-*(*α*-l-rhamnopyranosyl)-3,3',4-tri-*O*-methylellagic acid (**3**).


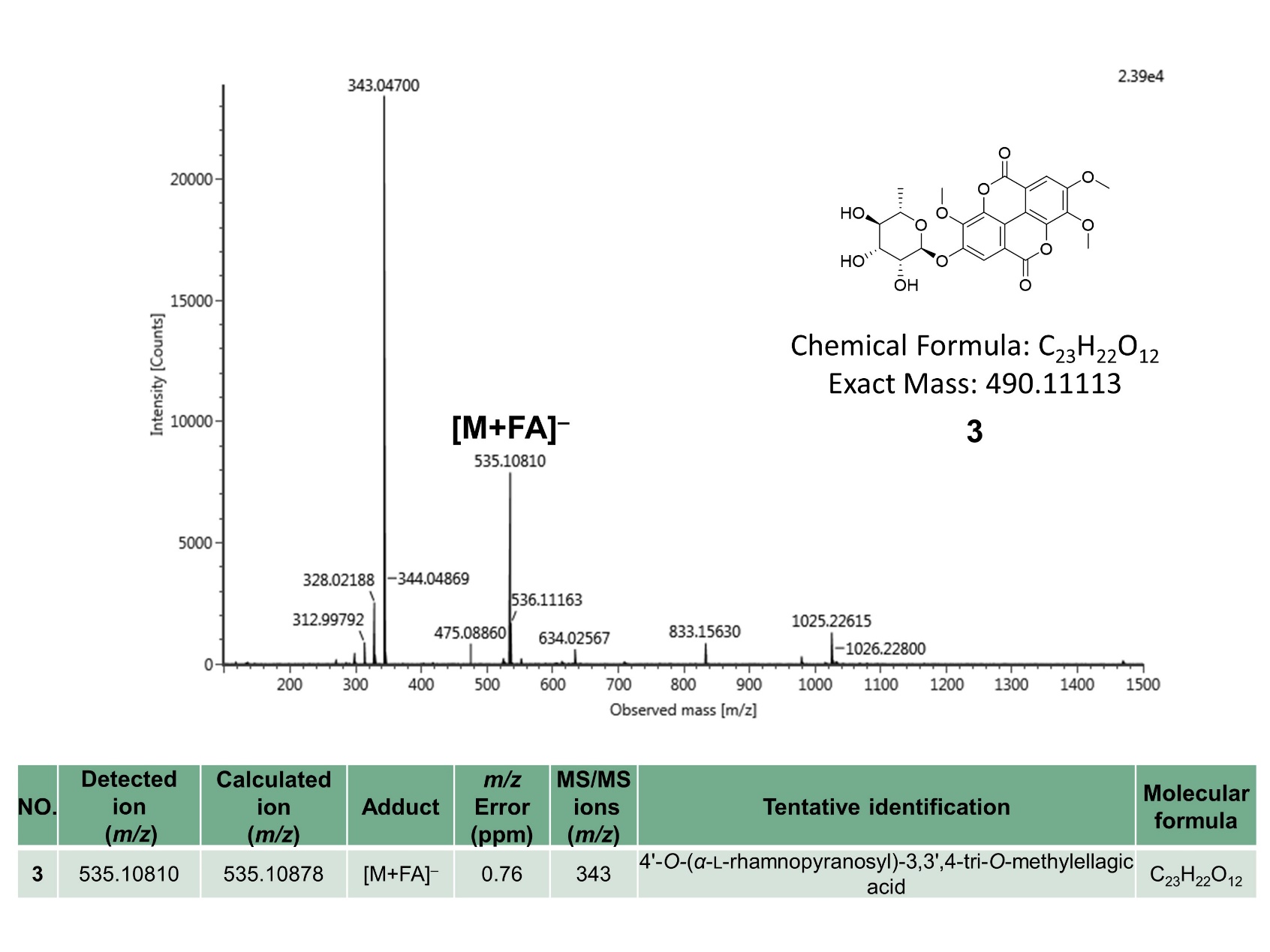


**Figure S6.** HR-ESI-MS spectra of 4'-*O-*(*α*-l-rhamnopyranosyl)-3,3',4-tri-*O*-methylellagic acid (**3**).


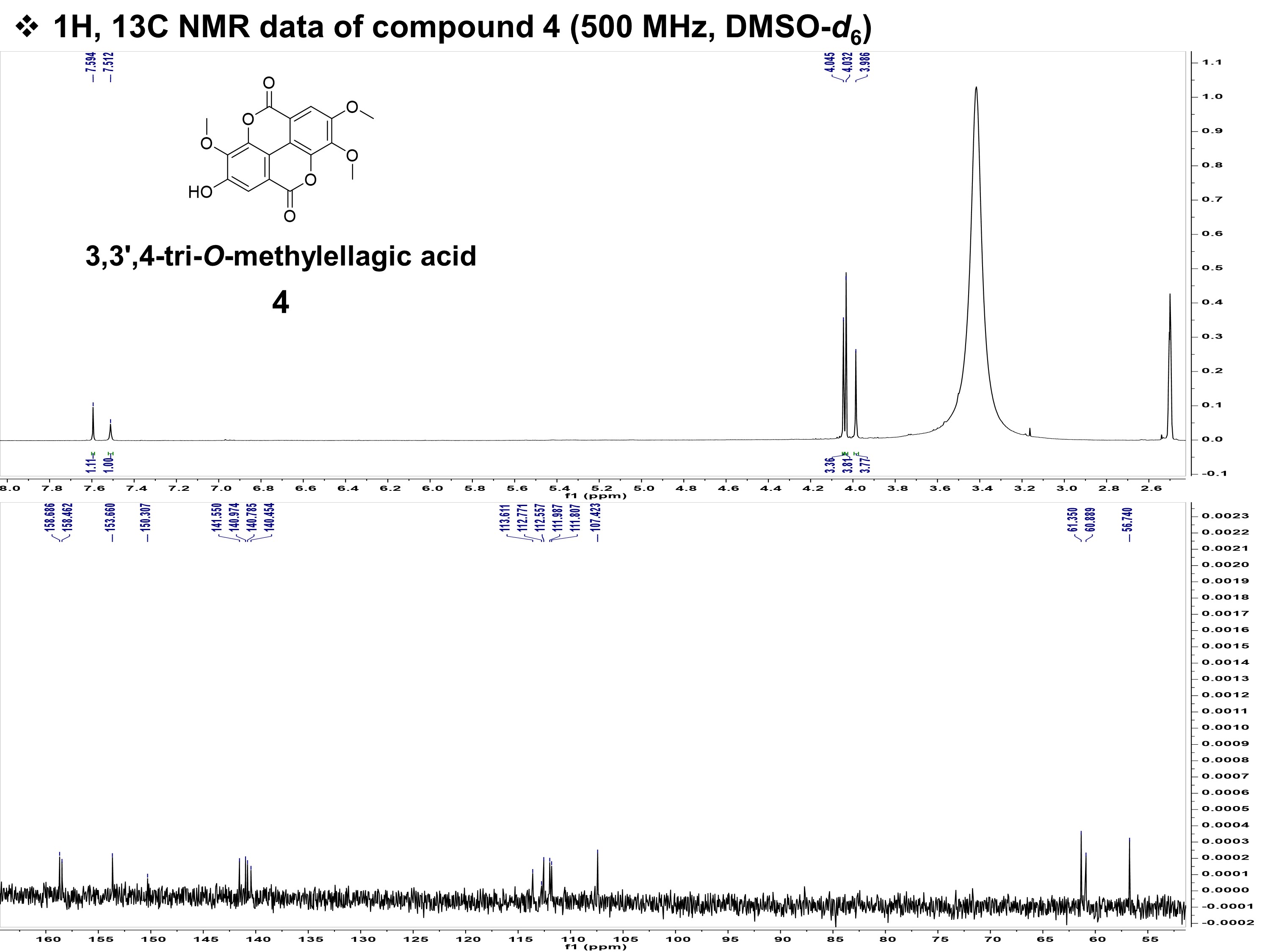


**Figure S7.** ^1^H and ^13^C NMR (500 and 100 MHz, DMSO-*d_6_*) spectra of 3,3',4-tri-*O*-methylellagic acid (**4**).


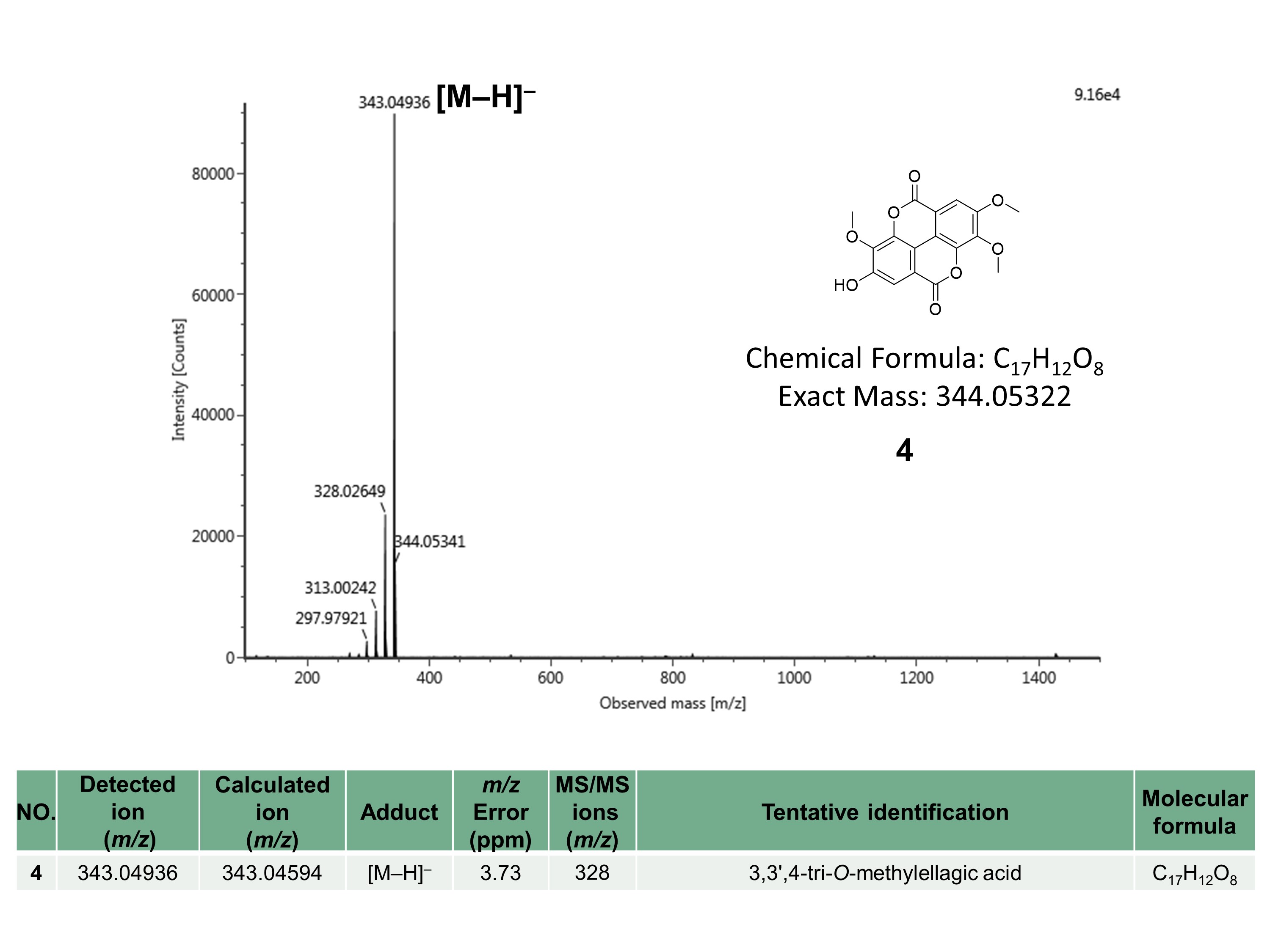


**Figure S8.** HR-ESI-MS spectra of 3,3',4-tri-*O*-methylellagic acid (**4**).


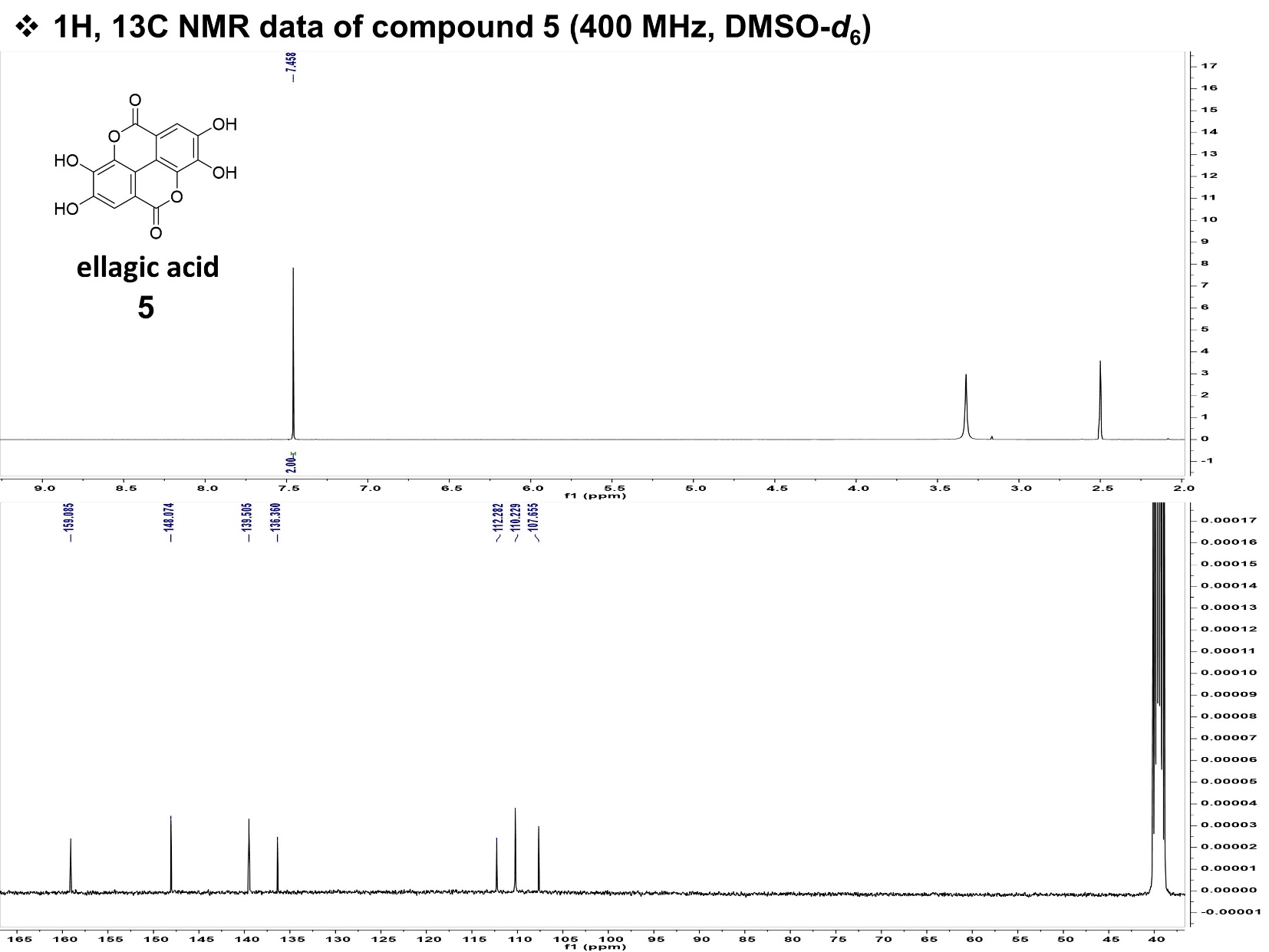


**Figure S9.** ^1^H and ^13^C NMR (400 and 100 MHz, DMSO-*d_6_*) spectra of ellagic acid (**5**).


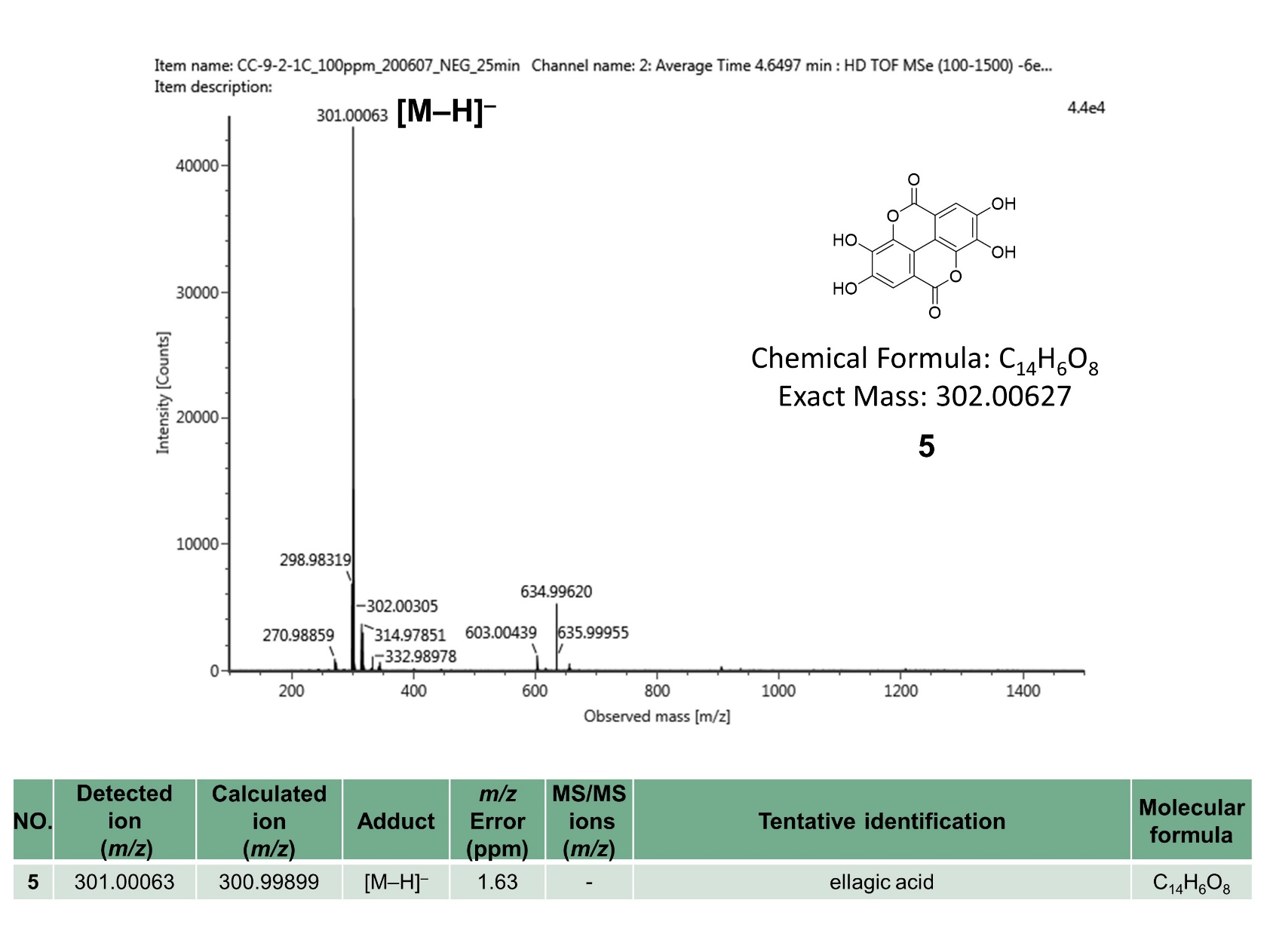


**Figure S10.** HR-ESI-MS spectrum of ellagic acid (**5**).


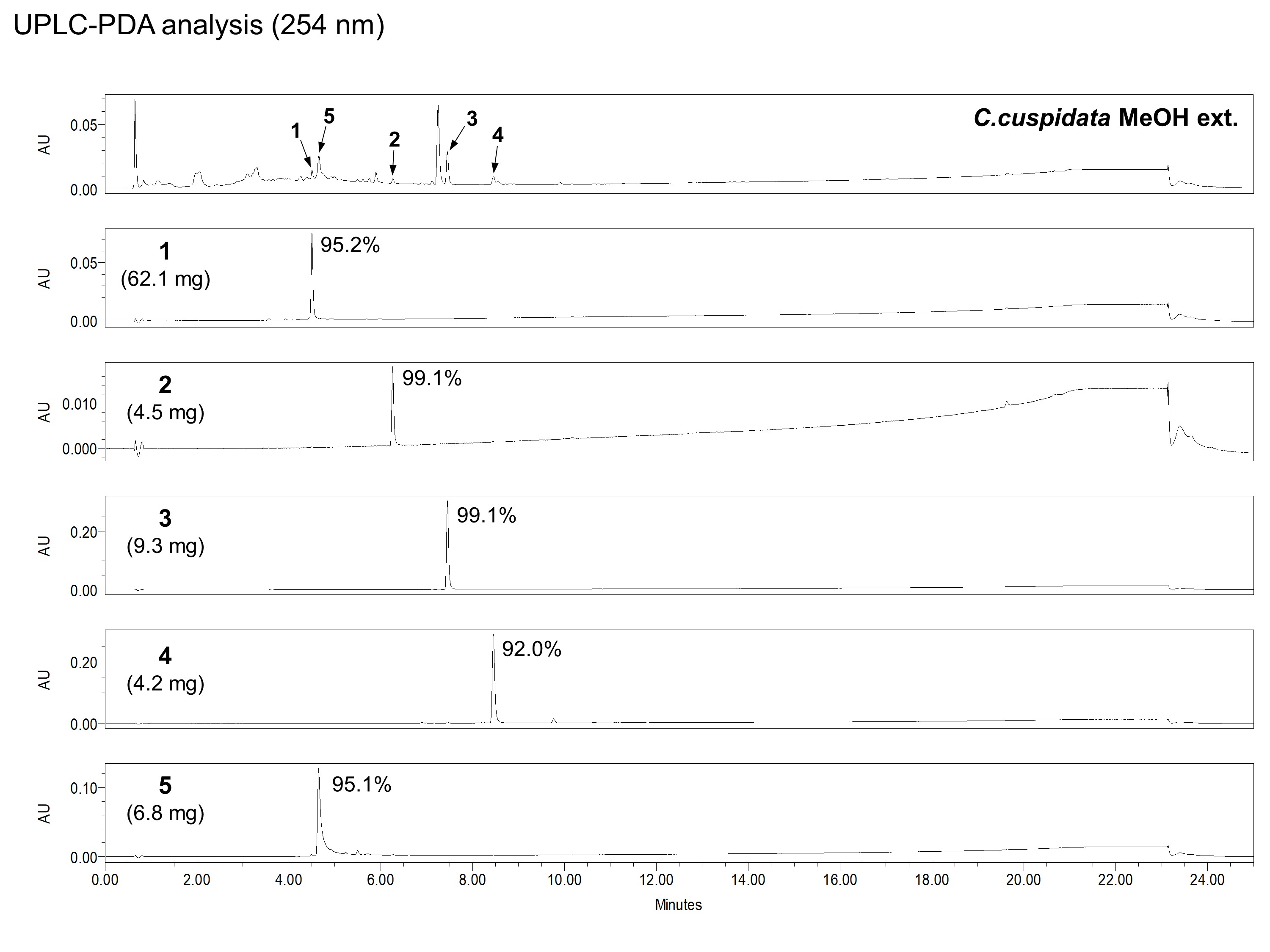


**Figure S11.** The purities of the isolated compounds **1**–**5**.
